# The Influence of Water Deficit on Dehydrin Content in Callus Culture Cells of Scots Pine

**DOI:** 10.3390/plants13192752

**Published:** 2024-09-30

**Authors:** Natalia Korotaeva, Vladimir Shmakov, Vadim Bel’kov, Daria Pyatrikas, Sofia Moldavskaya, Igor Gorbenko

**Affiliations:** Siberian Institute of Plant Physiology and Biochemistry, Siberian Branch of the Russian Academy of Sciences, Irkutsk 664033, Russia; vladwork70@gmail.com (V.S.); anvad.irk@rambler.ru (V.B.); galdasova@sifibr.irk.ru (D.P.); matmod@sifibr.irk.ru (S.M.); gorbenko@sifibr.irk.ru (I.G.)

**Keywords:** callus culture, water deficit, dehydrins, *Pinus sylvestris* L.

## Abstract

Under a water deficit, the protective proteins known as dehydrins (DHNs) prevent nonspecific interactions in protein and membrane structures and their damage, in addition to playing an antioxidant role. The DHNs of a widespread xerophytic species Scots pine (*Pinus sylvestris* L.) have been poorly studied, and their role in resistance to water deficits has not been revealed. In this paper, we have expanded the list of DHNs that accumulate in the cells of Scots pine under the conditions of water deficits and revealed their relationship with the effects of water deficits. In this investigation, callus cultures of branches and buds of Scots pine were used. A weak water deficit was created by adding polyethylene glycol to the culture medium. Under the conditions of a water deficit, the activity of catalase and peroxidase enzymes increased in the callus cultures. A moderate decrease in the total water content was correlated with a decrease in the growth rate of the callus cultures, as well as with an increase in the activity of lipid peroxidation. The accumulation of Mr 72, 38, and 27 kDa DHNs occurred in the callus cultures of buds, and the accumulation of Mr 72 and 27 kDa DHNs positively correlated with the lipid peroxidation activity. An increase in the content of DHNs was observed in cultures that differed in origin, growth indicators, and biochemical parameters, indicating the universality of this reaction. Thus, previously undescribed DHNs were identified, the accumulation of which is caused by water deficiency and is associated with manifestations of oxidative stress in the kidney cells of Scots pine.

## 1. Introduction

Drought is a widespread natural factor limiting plants’ growth and development both in natural environments and in the anthropogenic activities involved in crop production. Increasing climate aridification [1] makes the study of the effects of droughts on plants especially relevant. The main consequence of soil drought is a lack of water in plants. At the physiological level, this state leads to a slowdown in growth and photosynthesis, oxidative stress development [2], and changes in respiration and transport modes [2]. Drought tolerance is one of possible plant water deficit (WD) responses. Osmoprotective, antioxidant, and detoxification activities are the basis of this response at the cellular level [2]. The protective proteins known as dehydrins (DHNs) function as osmoprotectors and antioxidants and play an important role in the development of WD tolerance.

DHNs belong to group II of the LEA (late embryogenesis abundant) protein family [3] and also to the intrinsically disordered proteins group [4]. A structural feature of all DHNs is the presence of a lysine-rich K-sequence at the C-terminal region of the molecule. The presence of a K-sequence, as well as S-, Y-, and F-sequences, which are combined and duplicated at different positions of different DHNs, allows them to bind to membrane sites, proteins, nucleotides, metal ions, and reactive oxygen species (ROS) molecules [3,5,6]. Due to the variability in their molecular conformation, DHNs can act as a “molecular shield” for proteins and as membrane protectors, preventing unwanted interactions of these structures under physiological stress conditions [7]. The ability of DHNs to reduce the free ROS content during osmotic stress periods contributes to the increase in osmotic stress resistance [8]. DHNs accumulate in response to the most common stressors including water deficits [3,9]. The differential activity of DHN genes in various organs [10,11] and in response to various influences [11,12] is well known. The same DHNs can be involved in protective responses to different stressors including water deficits [12]. When several external factors act on a plant simultaneously under natural conditions, it is quite difficult to identify a specific relationship between the impact and the accumulation of a particular DHN. It is also difficult to identify such a connection in woody plants, since they have broad compensatory capabilities to reduce the negative effects of external factors.

The protective mechanisms of coniferous trees that respond to moisture deficiency are being actively studied [13]. Recently, the data on DHNs in coniferous plants have been expanded significantly, especially those on the expression of genes encoding these proteins [14,15,16,17]. The group-specific features of gymnosperm DHN protein structures have been established, which contain A- and E-sequences and lack the Y repeat that is typical for angiosperm DHN proteins [15]. The DHNs of the species *P. sylvestris* are among the least studied [18,19,20]. At the same time, most studies of the dehydrins of *P. sylvestris* are devoted to the genes of these proteins [21,22,23,24]. Only a few studies have focused on the response of *P. sylvestris* DHNs to water deficiency at the protein level although this tree belongs to the xerophyte group; therefore, it seems reasonable to determine the mechanisms for combating water deficiency in Scots pine. The accumulation of major DHNs with a mass of 65 kDa possessing the A-, E-, K-, and S-sequences was found to be correlated with the degree of WD [25]. We believe that Scots pine can accumulate more than a single DHN in response to water scarcity. Pine dehydrins with masses of 45 and 50 kDa were additionally identified in water deficiency conditions [25]. However, despite the fact that the authors used low and high levels of water deficiency, these 45 and 50 kDa DHNs were present in a few samples and were absent in others; therefore, they were not considered further [25]. We assume that the reason for the detection of accumulation in response to an aqueous deficiency of a single dehydrin of 65 kDa in Scots pine [25] may be the use of seedlings, since the xerophytic plant is able to successfully compensate for water deficiency. In our opinion, the identification of more dehydrins that are associated with water deficiency will be facilitated by the use of a plant cell model in which it is impossible to compensate for the effects of water deficiency using the resources of the whole plant. We believe that callus cultures are suitable for this purpose. Another unsolved problem is the identification of the exact functions of coniferous dehydrins under the action of water deficiency. As mentioned earlier, dehydrins are able to interfere with non-specific interactions of membranes and proteins. They also utilize reactive oxygen species (ROS), the content of which increases under the action of water deficiency [3]. We assume that the utilization of ROS may be the main purpose of pine dehydrins under the action of a weak water deficit. The aim of the study was to identify several DHNs accumulating in the cells of the callus culture of Scots pine in response to a mild water deficiency and also to check whether these proteins are associated with the development of the oxidative stress that accompanies water deficiency. To achieve these goals, callus cultures of shoots and buds of Scots pine were obtained. The callusogenesis and obtained cultures were characterized, which were then exposed to a mild water deficiency. After that, the changes in the indicators of oxidative stress and the content of dehydrins were evaluated and the links between the listed parameters were revealed.

## 2. Results

### 2.1. Growth Characteristics of Callus Cultures

**Figure 1:**
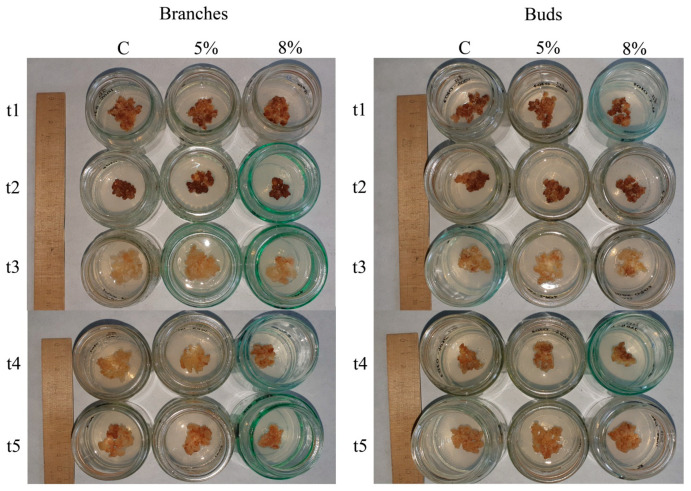
The appearance of PEG-treated (5 and 8%) and control callus samples. A typical appearance is presented. C—control (untreated); t1, t2, t3, t4, and t5 represent the numbers of individual trees.

Callus cultures are not a generally accepted subject for studying stress reactions, so we have described in detail the receipt of the cultures and the features of their growth. Plant material (buds and apical shoots) cut from five Scots pine trees was used to obtain callus cultures of their buds and branches (Figure 1). To evaluate the physiological differences between callus cultures of different origins (induced from branches or buds) and different genotypes (obtained from individual trees), we examined the growth and biochemical parameters of callus cells. The resulting callus cultures had mainly a loose consistency. The calli obtained from the t1, t3, t4, and t5 explants were light yellow in color, sometimes with brown spots. The calli from the t2 explants were mostly brown. The process of obtaining the callus cultures and the appearance of the obtained calli are shown in Figures S1 and S2.

To characterize the subject of this study, we evaluated the process of callusogenesis (Tables S1–S3) and the growth and viability characteristics of the obtained callus cultures (Table 1, Table 2 and Table 3). The analysis of callusogenesis showed differences in this process both between explants obtained from different trees and explants of different origin (branches or kidneys) (Tables S1–S3).

A significant level of heterogeneity in the callus growth rate (CGR) values was found both among individual trees and the plant material cut from each tree, as well as between the dates of the growth rate assessments (Table 1 and Table 2).

The highest CGR values by day 15 of cultivation were detected on derivatives of the t3 bud explants. By day 30 of the cultivation period, the maximum CGR was detected in the derivatives of branches of the t4 and t3 explants, the growth of which was found to be 1078% and 674%, respectively, for 15 days of cultivation. The CGR was also the greatest for derivatives of the branches of the t3 explants in the period from 30 to 45 days and from 45 to 60 days of cultivation, compared to other derivatives of explants (the growth was 398% in the period from day 30 to day 45 and 269% from day 45 to day 60 of cultivation).

The viability of the callus cultures (VC) was assessed by the timing of the first appearance of necrotization signs and by the timing of the cultures’ death over the 4-month incubation period (Table 3). Areas of necrosis during the cultivation process were found on the plant material of all five trees. The earliest appearance of necrotic areas was detected in the culture obtained from the buds and branches of the t1 explant and the buds of the t2 explant (at 2–3 weeks of cultivation). The necrotization of culture cells obtained from the buds and branches of the t5 explant and the branches of the t4 explant occurred much later. The cultures obtained from the t1 and t2 trees were characterized by their complete death within 4 months of the cultivation period. Thus, over 4 months of cultivation, complete death was recorded in 15% of the calli obtained from the bud explants of t1, 24% of the calli obtained from the branch explants of t1, 4% of the calli from the buds of t2, and 27% of the calli from the branches of t2. The analysis of callusogenesis showed that the callus cultures obtained from different trees and different origins differed significantly in growth and development parameters.

### 2.2. Physiological and Biochemical Parameters of Callus Cultures under Control and Stress Conditions

**Figure 2:**
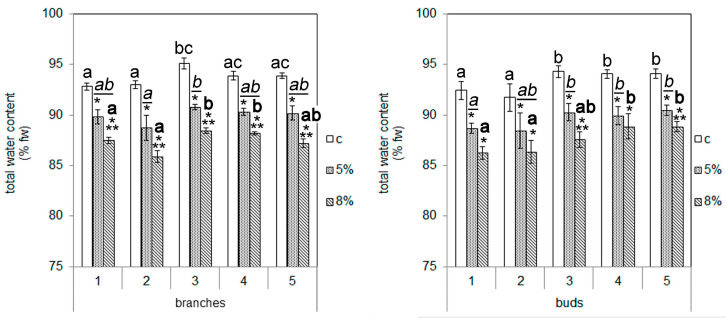
The influence of PEG concentrations on total water content in cells of callus cultures. The digits represent numbers of the trees that were used as sources of plant material for callus induction. The means and standard errors of the mean are shown (n = 3–6). * indicates significance differences among control (c) and treatment (5 or 8% PEG); ** indicates significance differences among treatments (*p* < 0.05). Different letters indicate significance differences among calli obtained from different trees at *p* < 0.05 (the regular font corresponds to control; the underlined italic font corresponds to 5% PEG; the bold font corresponds to 8% PEG). fw—fresh weight.

The water content of the t1 and t2 calli at control conditions was lower than in the other callus cells (Figure 2). The addition of 5 or 8% PEG to the culture medium led to a moderate dose-dependent reduction in the total water content in the cells of all studied calli, which did not exceed 3–4% at 5% PEG and 6–7% at 8% PEG. After the addition of PEG, the inter-cultural differences in the total water content remained constant, and the lowest water content was in the cells of the calli of the t1 and t2 explants.

To detect if a mild water deficiency affected the callus cultures, the growth and biochemical indexes of the calli were investigated under control and water deficit conditions.

In all studied cultures, except for t2 (branches), the addition of PEG to the culture medium caused a decrease in the callus growth rate (CGR) (Table 4). The magnitude of growth rate suppression depended on the dose of added PEG. PEG at a concentration of 5% had approximately the same effect on the growth rate of calli induced from the branches of t1, t3, and t4 and the buds of t4. The decrease in growth rate in all cases was found to be within 7–9% of that of the control. In the case of the cell cultures obtained from the buds of the t4 explants, the effect of PEG at a concentration of 8% did not exceed the effect on the growth rate of the 5%-PEG-treated calli. At the same time, 8% PEG significantly reduced the growth rate of the calli—the derivatives of the branches of t3 and t4 and the buds of t5. PEG at a concentration of 8% had the greatest negative effect on the growth rate of the cultures obtained from the buds of the t3 explant and the branches of t1, which was expressed as a 20% decrease in the growth rate compared to that of the control samples. Calli obtained from the t2 tree were characterized by the lowest growth rate under the control conditions (145% and 133% growth for branches and buds, respectively). In the case of using material from t2 branches, no significant effect of 5 or 8% PEG on the growth rate was detected. At the same time, for the calli from the buds of the t2 explants, the growth rate in presence of 5% PEG decreased by 12%, and in presence of 8% PEG, it decreased by 17% in relation to the control. The presence of 5% PEG in the culture medium reduced the growth of the calli obtained on the branches of the t5 explants slightly (within 3% of the control values). And the decrease in the rate of callus growth in the case of the material taken from the buds of the t1 explant on media containing PEG, regardless of its concentration, was approximately 10%. Thus, the WD had a rather heterogeneous effect on the growth rate of the callus cultures. The results obtained do not allow us to clearly characterize the individual genotypes used in the current study in terms of the sensitivity of the culture growth to WD and require more detailed studies using other methods. But the investigation of the CGR without PEG and after its use allows us to conclude that a low level of water deficiency prevents the growth of callus cultures.

**Figure 3:**
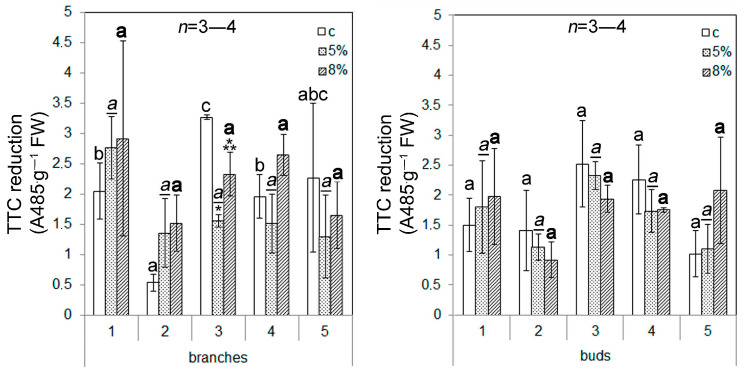
The influence of water deficit (WD) on viability of callus cell cultures. The digits represent numbers of the trees that were used as sources of plant material for callus induction. The means and standard errors of the mean are shown (n = 3). * indicates significance differences among control (c) and treatment (5 or 8% PEG); ** indicates significance differences among treatments (*p* < 0.05). Different letters indicate significance differences among calli obtained from different trees at *p* < 0.05 (the regular font corresponds to control; the underlined italic font corresponds to 5% PEG; the bold font corresponds to 8% PEG). FW—fresh weight.

The assessment of the viability of callus culture cells (VCC) based on the rate of TTC reduction makes it possible to identify the dehydrogenase activity of cells and examine their enzymatic activity. Under the control conditions, the highest VCC values of the branches of the callus cultures were observed for the t3callus cells; the lowest VCC values were observed for the t2 callus cells (Figure 3). The VCC remained unchanged under WD in almost all of the tested callus cultures, except for the calli of the t3 branches (the VCC was decreased). Otherwise, we did not identify the effect of the doses of PEG used on the viability of the callus cultures.

Among the parameters characterizing intracellular oxidative processes, the concentration of hydrogen peroxide (HP) and the superoxide anion (SA), the activities of the catalase (CAT) and peroxidase (POD) enzymes, and the activity of lipid peroxidation (LP) were studied in the calli cells.

**Figure 4:**
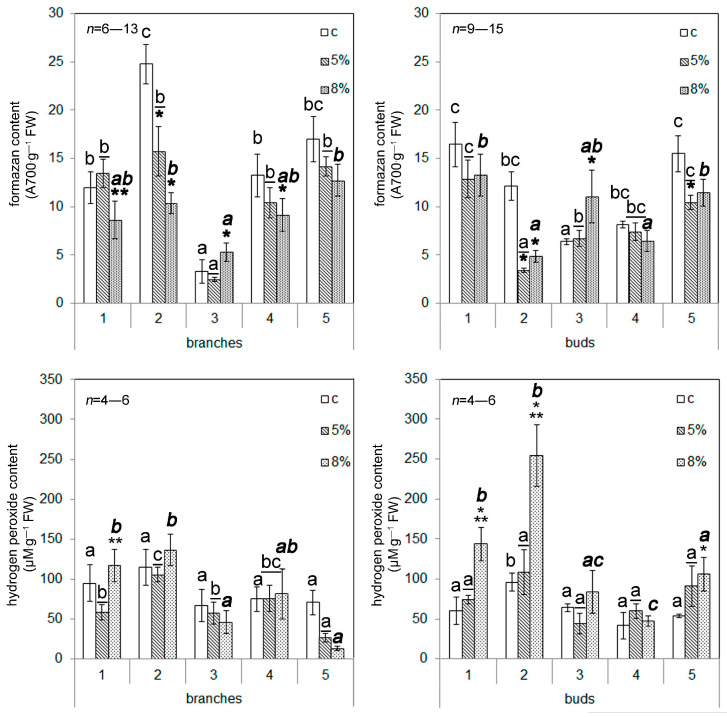
The influence of water deficit (WD) on hydrogen peroxide content and superoxide anion content in callus culture cells. The digits represent numbers of the trees that were used as sources of plant material for callus induction. The means and standard errors of the mean are shown. * indicates significance differences among control (c) and treatment (5 or 8% PEG); ** indicates significance differences among treatments (*p* < 0.05). Different letters indicate significance differences among calli obtained from different trees at *p* < 0.05 (the regular font corresponds to control; the underlined italic font corresponds to 5% PEG; the bold font corresponds to 8% PEG). FW—fresh weight.

An increased superoxide anion (SA) content under the control conditions was noted for the t2 samples of callus cells (branches), as well as the t1 and t5 samples (buds); the lowest SA content was observed for the t3 samples (branches), as well as the t3 and t4 samples (buds) (Figure 4). The PEG addition led to a decreased SA content in the t2 and t4 callus cells (branches) and the t2 and t5 callus cells (buds). An increased SA content in response to WD was observed in the t3 sample’s branches and buds. There were no significant differences in hydrogen peroxide (HP) content between the samples of different origins under the control conditions, except for the increased HP content in the t2 cells (buds) (Figure 4). The addition of PEG influenced the HP content in cells of limited callus samples. The HP content was increased in cells of the t1, t2, and t5 samples (buds). Thus, a slight decrease in the total water content did not lead to significant changes in the content of reactive oxygen species or the viability of the callus cells, although a decrease in the growth rate of calli was observed.

**Figure 5:**
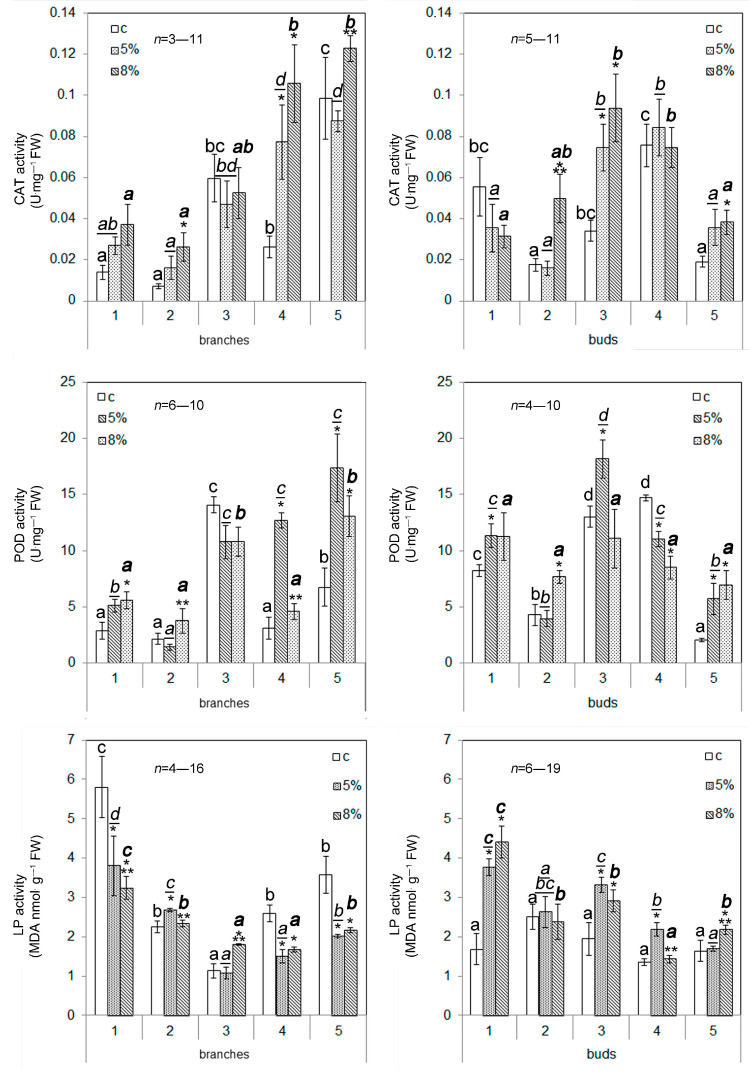
The influence of water deficit (WD) on CAT, POD, and LP activities in cells of callus cultures. The digits represent numerical indexes of the trees that were used as sources of plant material for callus induction. The means and standard errors of the mean are shown. n = 3. * indicates significance differences among control (c) and treatment (5 or 8% PEG); ** indicates significance differences among treatments (*p* < 0.05). Different letters indicate significance differences among calli obtained from different trees at *p* < 0.05 (the regular font corresponds to control; the underlined italic font corresponds to 5% PEG; the bold font corresponds to 8% PEG). FW—fresh weight.

Under the control conditions, the highest lipid peroxidation (LP) activity among the calli obtained from buds was recorded in the t2 sample; among the cultures obtained from branches, the LP activity was low in the t3 sample and increased in the t1 and t5 samples (Figure 5). After the addition of PEG to the culture media, the t2 and t3 cell cultures (branches) and t1, t3, t4, and t5 cultures (buds) were found to have increased LP activity. The differences between cultures with increased LP activity were expressed as the increase in LP in response to the 5% PEG treatment (the t2 branches and t4 buds) or in response to the 8% PEG treatment (the t3 branches and t5 buds), or the calli in which both PEG doses had the same effect (the branches of t4 and t5 and the buds of t1 and t3). The decrease in LP activity under WD was recorded in the branches of the t1, t4, and t5 callus cultures. In the t2 cells (buds), changes in LP activity were not observed.

Under the control conditions, the activity of catalase (CAT) and peroxidase (POD) in the t3 and t5 cell samples (branches) and in the t1, t3, and t4 samples (buds) was higher than in other samples (Figure 5). The differences in CAT activity between the callus cells of different origins were exactly the same as those for the level of POD activity. The exceptions were the samples of the t3 callus cells (buds) and the t5 cells (branches), in which the POD activity or CAT activity, respectively, were found to be higher. The activity level of antioxidant enzymes under the influence of WD underwent changes, which differed for calli of different origins and different genotypes. The level of CAT activity in response to PEG treatment increased in more than half of the tested calli (Figure 5). In the t3 and t5 calli (branches) and t4 calli (buds), the CAT activity remained unchanged. The POD activity increased in response to the addition of 5 or 8% PEG to the growth medium in most of the callus cultures studied. A decrease in POD activity occurred in the t4 callus cells induced from buds.

From this investigation of the growth and biochemical alterations of callus cultures under weak water deficit conditions, we can conclude that shifts in reactive oxygen species (ROS) content and viability were minimal. The same cannot be said about measurements of lipid peroxidation, growth, or enzyme activity.

A correlation analysis was used to assess the relationship between the measured physiological parameters. When 5% PEG was added to the calli (branches), the total water content had a close inverse correlation with the level of lipid peroxidation activity (R = −0.706; *p* = 0.037) (Table S5), which was not observed under the control conditions (Table S4). When 8% PEG was added to this group of cultures, the growth rate became strongly correlated with viability (R = 0.9; *p* = 0.037) (Table S6). Both in the control and under the action of 5% PEG, the growth rate of the buds of the callus cultures was closely negatively related to the total water content (R = −0.857, *p* = 0.0048 for the control; R = −0.927, *p* = 0.037 for the group with 5% PEG). A negative correlation between lipid peroxidation and the total water content appeared when 8% PEG was added to the buds of the callus cultures (R = −0.749; *p* = 0.037) (Table S6). A strong negative relationship was observed between a decrease in total water content and a decrease in the growth of the calli of branches under the action of 8% PEG (R = −0.813; *p* = 0.037), as well as between a decrease in growth and a shift in peroxidase activity for the calli of buds when exposed to 5% PEG (R = −0852; *p* = 0.037) (Table S7). The assessment suggests that a decrease in growth, an increase in peroxidase activity, and lipid peroxidation in callus cells are closely associated with a decrease in total water as a result of the action of PEG.

### 2.3. The Impact of Water Deficit on DHN Gene Activity and DHN Protein Accumulation

**Figure 6:**
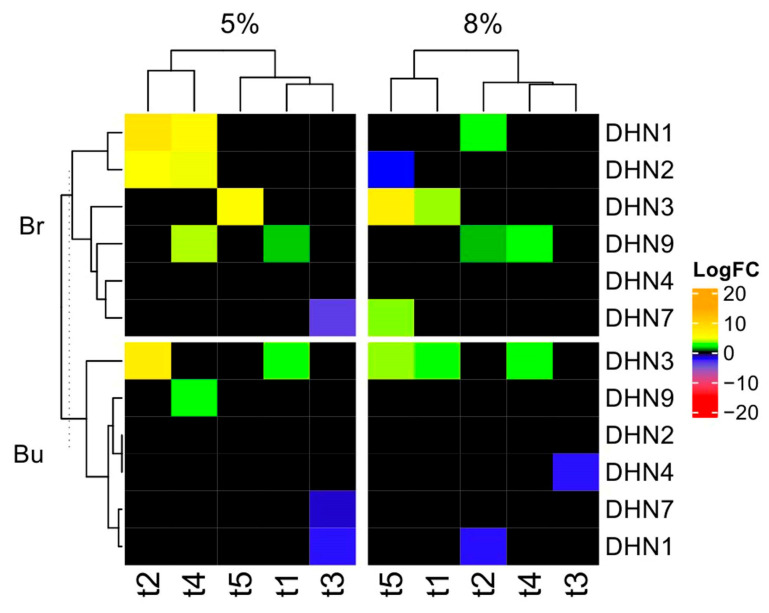
Heatmap of DHN gene expression log_2_-fold changes (LogFC) in calli under PEG treatment in relation to control conditions. Br—sample calli initiated from branch tissues; Bu—sample calli initiated from bud tissue; 5% and 8% are percentages of PEG added to growth media for WD induction. t1–t5 represent different trees used for callus culture initiation. Three independent biological replicates were used, and only results with significant expression differences (*P*_t-test_ < 0.05) from control conditions are shown. *ACT-1* expression is used for normalization.

The RT-qPCR method was used to compare the transcript levels of DHN genes in the calli induced from branches and buds cut from five different Scots pine trees under control conditions in relation to the *ACT-1* transcript level (Figure 6). Primers for eight DHN genes were used, only six of which allowed us to obtain the corresponding PCR products: *DHN1*, *DHN2*, *DHN3*, *DHN4*, *DHN7,* and *DHN9* (see Materials and Methods).

After the 5% PEG treatment, *DHN1*, *DHN2*, *DHN3,* and *DHN9* transcript levels were found to be significantly upregulated in the calli initiated from the Scots pine branches of the following trees: t1 and t2 (*DHN1* and *DHN2*), t4 (*DHN1*, *DHN2*, and *DHN9*), and t5 (*DHN3*). At the same time, the *DHN3* transcript in the t3 branches’ calli was found to be downregulated significantly (Figure 6). The t4 calli had the biggest number of expressed genes of DHN during the water deficit (WD). The addition of 8% PEG to the growth media of the branches’ calli resulted in the upregulation of *DHN1* (t2), *DHN3* (t1 and t5), *DHN7* (t5), and *DHN9* (t2 and t4), as well as the downregulation of *DHN2* (t5). The transcript levels of DHNs in the calli of the t3 branches remained unchanged.

In the buds’ calli, the addition of 5% PEG to the growth medium resulted in the upregulation of *DHN3* (t1 and t2) and *DHN9* (t4) compared to the control, as well as the downregulation of *DHN7* and *DHN1* (only in the t3 calli). The addition of 8% PEG to the growth medium resulted in the upregulation of *DHN3* (t1, t4, and t5) and the downregulation of *DHN1* (t2) and *DHN4* (t3).

Thus, the greatest number of genes responding to the WD was observed in the calli samples initiated from the branches of Scots pine, but not from the buds. We found that the calli induced from the branches respond to the PEG treatment with the upregulation of three genes (*DHN1*, *DHN3,* and *DHN9*), while the upregulation or downregulation of two genes (*DHN2* and *DHN7*) was found to depend on the intensity of treatment. The calli induced from buds demonstrated the upregulation of two genes (*DHN3* and *DHN9*) and the downregulation of three genes (*DHN1*, *DHN4,* and *DHN7*) in response to the WD. We did not observe any significant changes in *DHN4* transcript level in the branches’ calli or in *DHN2* in the buds’ calli. In the calli of t3, only the downregulation of the transcripts of dehydrin genes was observed.

**Figure 7:**
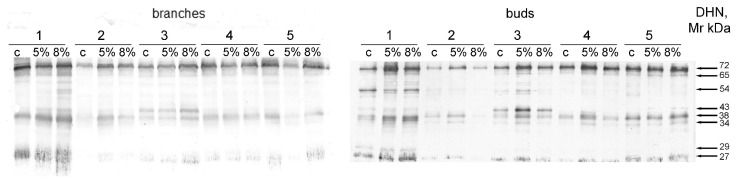
The influence of water deficit (WD) on DHN accumulation in cells of callus cultures. The digits represent numerical indexes of the trees that were used as sources of plant material for callus induction. c—control; 5%—5% PEG; 8%—8% PEG. The digits on the right represent the molecular weights of the detected DHNs. Typical membranes are presented.

Gel electrophoresis followed by Western blotting and the use of specific antibodies made it possible to identify eight DHNs in the callus cultures growing under the control conditions. These were DHNs with masses of 72, 54, 47, 43, 38, 34, 29, and 27 kDa (Figure 7). Among the listed DHNs, only proteins with masses of 72, 47, 38, and 27 kDa were detected in all of the studied samples. The 43 kDa DHN was clearly detected only in the t3sample (branches and buds), and the 54 kDa DHN was detected in the t1 cells and, to a lesser extent, in the cells of t3 and t5 (buds) and those of t3 and t4 (branches).

**Figure 8:**
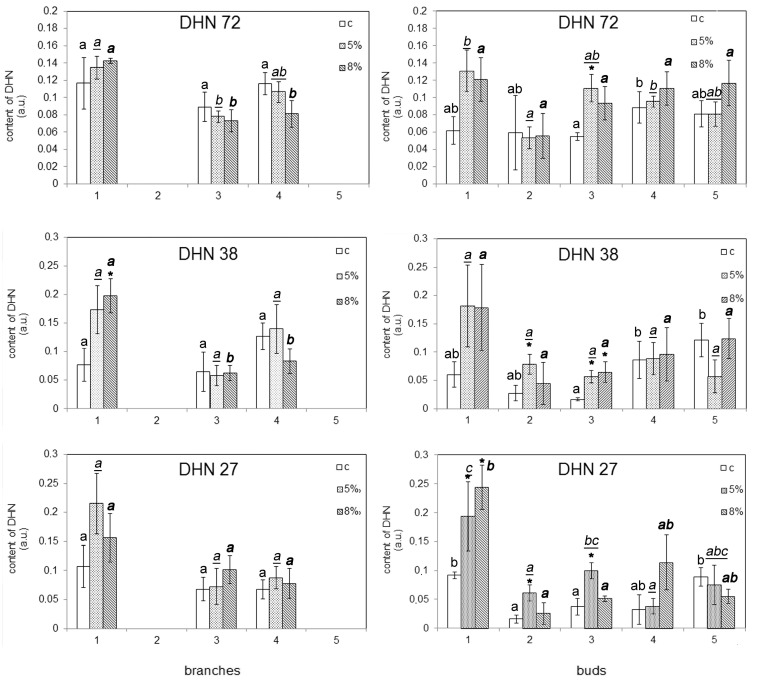
The influence of water deficit (WD) on the content of 75, 38, and 27 kDa DHNs in cells of callus cultures. The digits represent numbers of the trees that were used as sources of plant material for callus induction. The means and standard errors of the mean are shown. n = 3–4. * indicates significance differences among control (c) and treatment (5 or 8% PEG) (*p* < 0.05). Different letters indicate significance differences among calli obtained from different trees at *p* < 0.05 (the regular font corresponds to control; the underlined italic font corresponds to 5% PEG; the bold font corresponds to 8% PEG). The data on the DHN content of cells of t2 and t5 branches were obtained in duplicate and are not shown in this diagram.

After the addition of PEG, DHNs with masses of 72, 38, and 27 kDa were detected in all of the studied samples. The content of these proteins was found to be increased under the water deficit (WD) in the callus cultures of the buds of t1, t2, and t3 (except for DHN Mr 72 kDa at t2) (Figure 8). An increase in the content of DHN Mr 27 kDa was also recorded for t4. At the same time, the accumulation of dehydrins Mr 72 and 27 kD in the callus cultures of buds under the action of 5% PEG was correlated with an increase in lipid peroxidation activity (accordingly, R = 0.952, *p* = 0.037; R = 0.812, *p* = 0.037) (Table S7).

Using the obtained data, we examined whether the protein amounts could be correlated specifically with the transcript levels; using a linear regression, we found that there is no reliable linear dependence between any of the protein amounts and measured transcript levels (including parameter–parameter interactions) (Figures S4–S6).

## 3. Discussion

In this study, the effect of moderate water deficiency on calli obtained from the explants of branches and buds of Scots pine was studied. The use of PEG caused a decrease in the total water content in the callus cells, which did not exceed 3–4% when using 5% PEG and 6–7% when using 8% PEG (Figure 2). At the same time, there were no changes in cell viability (Figure 3) (except for the t3 callus culture of branches) or in the reactive oxygen species (ROS) content (Figure 4) (except for the t3 callus culture of branches and the t2 and t3 callus cultures of buds) of the callus cultures. These results confirm that the effect of the water deficit on the callus cells was weak, and it had almost no effect on the listed indicators. The failure to detect the accumulation of ROS is obviously due to a shift in the balance between the ROS production and scavenging in the scavenging phase [26]. This became possible as a result of the mild level of exposure, which occurred for a long time (10 days). However, the growth of callus cultures (Table 4), lipid peroxidation activity, and the activity of catalase and peroxidase enzymes (Figure 5) changed under the influence of PEG.

In plants, one of the main stress indicators is growth arrest. A decrease in the callus growth rate in most of the callus cultures (except for the t2 callus culture of branches) indicates the physiological effect of the water deficit (WD) on the callus cultures. Additionally, when 5% PEG was used for the callus cultures of buds, a strong positive correlation was observed between the callus growth rate and the water content (Table S5) and between the alterations in these index values for the control and after the addition of PEG (Table S7). The increase in lipid peroxidation in response to the WD occurred in three branches’ callus cultures out of the five studied and in four of the bud cultures out of the five studied. Similarly, in most of the studied cultures, an increase in the activity of the antioxidant enzymes catalase and peroxidase occurred under the condition of a water deficit (Figure 5). An increase in the activity of these enzymes occurred even in the calli in which a decrease in lipid peroxidation activity was detected under the PEG treatment (t1, t4, and t5 of branches). The correlational analysis showed that a strong negative correlation between the total water content and lipid peroxidation rate appeared under the 5% PEG treatment of the cultures of branches (Table S5) and under the 8% PEG treatment of the cultures of buds (Table S6). The observed changes in the listed parameters indicate that a mild water deficit leads to an inhibition of the growth rate of callus cultures, enhances membrane oxidation, and causes an increase in the activity of antioxidant enzymes. An increase in the activity of lipid peroxidation and the enzymes catalase and peroxidase are associated with oxidative stress. Thus, the mild water deficit conditions that we used in the current study lead to the activation of oxidative stress in the callus cells.

Due to the water deficit, the accumulation of DHNs with masses of 72, 38, and 27 kDa occurred in the bud cultures (Figure 7 and Figure 8). The accumulation of dehydrins with masses of 72 and 27 kDa in the bud cultures in response to the water deficit was positively correlated with an increase in lipid peroxidation activity (Table S7), and the accumulation of 72 kDa DHN was correlated with an increase in peroxidase activity (Table S5).

Since the low level of water deficiency that we used leads to the activation of oxidative stress in the callus cells, and since the accumulation of dehydrins in the bud callus cultures as a result of the action of the water deficiency was positively correlated with an increase in lipid peroxidation activity, it can be assumed that the increase in dehydrin content is associated with the development of oxidation processes in the bud cultures. The importance of the accumulation of dehydrins may be to protect against the development of oxidative stress in the cells of buds’ callus cultures. It is known that dehydrins play a protective role in the action of oxidative stress [3,6]. The radical scavenging activity of dehydrins was suggested to be a result of the high content of amino acid residues susceptible to oxidative modification, such as glycine (Gly), histidine (His), and lysine (Lys), which were targets for radical-mediated oxidation in proteins [3]. The protection of cells from the adverse effects of ROS can be achieved through the interaction of DHN with heavy metals, since metal atoms can be a source of ROS as a result of stress [6].

In the current study, DHNs with masses of 72, 43, 34, 29, and 27 kDa were detected (Figure 7). DHNs with similar Mr values were previously found in Scots pine needles; changes in the amount of these proteins occurred depending on the season of the year [18,19,20]. The DHN with a mass of 38 kDa that was discovered in our work has not been previously detected in pine samples according to current data. DHNs with masses of 54 and 43 kDa also have not been detected previously. However, proteins with almost the same molecular masses have been identified in several samples of the needles and roots of *P. sylvestris*, but the authors detected these DHNs in the samples irregularly, so they did not study these proteins [25]. The detection of these DHNs in the callus cells in our study can be explained by significant quantitative differences in the accumulation of these proteins in callus culture cells and in the tissues of the tree as well as by a change in the cascade of signals in dedifferentiated cells compared to the level of the organism.

Almost all of the detected DHN proteins, except for that with a mass of 34 kDa, were detected in the callus cells under the control conditions (Figure 7). During the period of callus culture induction in the cell dedifferentiation process, a change in gene expression levels occurs [27,28]. Such a change may be a possible reason for the accumulation of DHN under the control conditions. The activation of cell dedifferentiation and the activation of DHN gene expression may have common transcription factors involved; e.g., auxin activates the NAC transcription factor, which is involved in the regulation of gene expression during the formation of dedifferentiated cells after wound stress [28]. The same regulator is involved in the signaling cascade of DHN gene expression during the period of salinity [5]. Although we have not found evidence in the literature, it is possible that dedifferentiation and the response of *DHN* gene expression to water deficiency have regulatory mechanisms in common.

In response to the WD, significant changes in transcript levels were recorded in the calli cells (Figure 6). We recorded significant transcript level changes in the *DHN1*, *DHN3,* and *DHN9* genes in the branches’ calli and *DHN3* and *DHN9* in the buds’ calli, suggesting the importance of these genes in response to a low level of WD. The transcript level of *DHN4* remains constant in response to WD, suggesting WD-associated signals do not participate in the regulation of the expression of this gene. The set of transcripts with altered expression was more considerable in the branches’ callus cells compared to the buds’ callus cells (Figure 6). This result is in contrast to the detected dehydrin accumulation in the calli of bud but not in the calli of branches (Figure 8). Using a linear regression, we found that there is no reliable linear dependence between any of the protein amounts and measured transcript levels (including parameter–parameter interactions) (Figures S4–S6). The regulation of DHN protein expression is a multi-stage process, controlled at both the transcript level and post-transcriptionally. The discrepancies between transcript and protein data are a common phenomenon [29]. The identification of coding genes corresponding to the identified dehydrins requires a separate study.

At the same time, the cultures induced from bud or branch explants differed in other parameters. For the bud cultures in our study, we detected a significant correlation between the growth rate of the callus culture and the viability of the callus culture cells (VCC) (R = 0.92, *p* = 0.027); for the branch cultures, the correlation was statistically insignificant (Figure S3). A low correlation force between the VCC and growth rate in the branch cultures may occur due to the high degree of heterogeneity in the cells of branch explants, which may include vascular, parenchymal, and cambial cells. Differences in gene expression induction in the branches’ and buds’ calli also indicate differences in the ability of cells of different origins to activate protective mechanisms in response to WD. A similar conclusion was reached assessing the functional characteristics of the genes active in the buds and needles of Scots pine using the Gene Ontology (GO) database: the enriched GO terms of bud transcripts were mainly associated with development-related processes as opposed to needle transcripts [22].

The analysis of callusogenesis parameters showed significant differences between Scots pine plant material cut from individual trees (genotypes). This result is in a good agreement with the data obtained in other studies aimed at studying callusogenesis in various species of higher plants. It is known that primary explants taken from plants of the same species, grown under the same conditions, do differ depending on the initial genotype (variety and line) in the frequency of callus formation and the intensity of growth of the cell culture [30,31]. Differences in characteristics of callusogenesis can frequently be detected using explants cut from the same genotype that differ in age and tissue [32,33].

The expression profiles of the dehydrin genes were different in the calli obtained from the different trees (Figure 6). Qualitative differences in DHN accumulation between callus cultures were observed (Figure 8). The presence of distinctions speaks in favor of genetically fixed differences in the DHN composition of these trees. Interindividual differences in DHN composition have previously been shown for Scots pine needles [20]. The identification of interindividual differences in the composition of dehydrins and of the variations in expression fold changes in response to WD in callus cultures obtained from different trees indicates the persistence of this phenomenon after obtaining a callus culture. It was previously shown that the features of the stress reaction of the initial plants can be traced in the callus cultures obtained from these plants [34,35]. But it is important to note that the dedifferentiation of cells that occurs during the production of a callus culture is accompanied by a change in the activity of the genes of the callus cells, which is the reason for cell fate transitions during callus formation in vitro and cellular reprogramming at the whole-genome level and which may limit the use of callus cultures in the study of stress resistance of adult plants [36,37].

The growth and physiological parameters that we studied in callus cultures of different genotypes (trees) and different origins (branches or buds) differed significantly even under control conditions. The WD caused changes in values of these parameters; these changes did not always have the same direction for different cultures. This observation indicates a significant contribution of genotypic features to WD response in calli cells. The growth characteristics and biochemical parameters varied in the callus cultures we obtained, both being assessed under the control conditions and after the stressor treatment. The current study allows us to identify genotypes that differed in growth and biochemical characteristics to the greatest extent. For the t2 callus culture under the control conditions, the low levels of viability and activity of the antioxidant enzymes CAT and POD, the increased level of lipid peroxidation, and the high ROS content indicate the low survivability of the callus cells. In the t3 and t4 cultures, the listed parameters had opposite values, which indicate the increased viability of these callus culture cells. Thus, the ability of the t2 culture cells to tolerate water deficits is weak, while the t3 and t4 culture cells could possibly be resistant to water deficits. This assumption is consistent with the literature data [38].

Under the control conditions, the content of dehydrins in the callus cultures of buds obtained from the different trees differed (Figure 8), which indicates possible differences in the studied genotypes’ response to water deficiency. According to the literature, differences in the content of dehydrins are indicators of stress resistance of trees of the genus Pinus [39,40]. At the same time, individuals tolerant to water deficiency pre-adapt to the fight against drought due to the constitutive accumulation of stress-related genes, which are found only in the late stages in sensitive individuals exposed to drought [40]. The magnitude of the dehydrin accumulation reaction in coniferous trees is also an indicator of cold resistance, while the activity of dehydrin accumulation in resistant trees is negatively regulated by enzymes and genetically fixed [39]. In this study, we did not determine the resistance of the callus cultures to water deficiency and did not compare such resistance with the resistance of experimental trees on the explants from which the callus cultures were obtained. Thus, there is only a limited possibility of projecting the information known from the literature on the causes of interindividual differences in the drought resistance of Scots pine trees, which is caused by dehydrins, to the reactions of the callus crops we have obtained. The elucidation of the mechanisms that cause interindividual differences in Scots pine trees in the reaction of dehydrin accumulation in response to water deficiency requires additional research.

On the other hand, in callus cultures of buds, regardless of the origin of the tissue type and the genotype of the tree used to obtain the callus, the contents of DHNs Mr 38 and 27 kDa were increased in response to the WD. The increase in content of the above-listed DHNs in the callus culture cells, which differ significantly in terms of growth rate and in the level of oxidative stress occurring during water deficit conditions, indicates the universality of such a response and suggests that the accumulation of these DHNs is not the result of a random reaction.

## 4. Materials and Methods

### 4.1. Plant Material, Callus Culture Initiation, Cultivation, and Stress Conditions

The use of seedlings, especially mature trees, can be an obstacle to the detection of dehydrins, which accumulate in response to non-destructive WD [25]. Therefore, in the study, we did not use plants, but rather callus cultures, which do not have the same compensatory capabilities as plant organisms. We used PEG concentrations in this study that caused a mild water deficiency that did not lead to the death of callus cells [41].

For callus culture production in the current study, five trees (t1–t5) of Scots pine (*Pinus sylvestris* L.) were used, growing in experimental field of the institute at which the research was conducted. This field was established with annual seedlings obtained at the forest nursery of the Meget locality in the region of the location of the institute in 1985. The soil on the field is gray forest, non-podzolic loamy type. Groundwater lies at a considerable depth (11–50 m) and does not have a noticeable effect on the soil moisture regime. The age of the trees used at the time of the study was 38 years. To obtain callus cultures, samples were collected in April 2023. The tops of branches from the lower third of the crown (approximately 3–4 cm) with needles and buds were taken as plant material for obtaining calli of Scots pine. The needles were removed prior to sterilization, and the buds with sections of bearing shoots were sterilized using sequentially a 3% solution of hydrogen peroxide (30 min) and a 0.1% solution of mercury chloride (10 min), then they were washed for 30 min in 3% hydrogen peroxide solution twice. To obtain explants, sterile buds with sections of bearing shoots were transferred to sterile filters (in Petri dishes). The total number of explants used in the work is presented in Table S1. Transverse disks that were 2–3 mm thick were cut out from the middle parts of the buds and bearing shoots, and the cuts were placed horizontally in a medium according to Murashige and Scoog [42] with modifications. Half of the medium was composed of macro- and microsalts with the addition of 0.8 mg·L^−1^ thiamine, 0.4 mg·L^−1^ pyridoxine, 0.4 mg·L^−1^ nicotinic acid, 100 mg·L^−1^ inositol, 200 mg·L^−1^ casein hydrolyzate, and 20 g·L^−1^ sucrose; 2,4-dichlorophenoxyacetic acid (2 mg·L^−1^) and 6-benzylaminopurine (0.5 mg·L^−1^) were used as growth regulators. Explants and the induced calli were cultured in the dark at a constant temperature of 25 °C. The duration of one cultivation cycle of resulting callus was 28 days. Before the start of the experiment, the period for growing callus cultures was two cultivation cycles. After this, to create the effect of WD, the calli were transplanted for 10 days onto a culture medium contained 5 or 8% polyethylene glycol 2000 (PEG, LOBA Chemie Fischamend, Fischamend, Austria), while the control samples were transplanted into a medium without the addition of PEG for the same period.

### 4.2. Callus Cultures’ Growth Characteristics

To characterize of the process of callus cultures undergoing callusogenesis was analyzed according to three criteria: (1) the callus induction rate (CIR)—which is the time needed for formation of first callus traces on the explant (days); (2) the frequency of callus induction (FCI)—which is the number of explants that formed callus, relative to the total number of explants used (%) (determined on the 2nd, 3rd, 5th, 10th, 15th, and 20th days of cultivation); and (3) the callusogenesis intensity (CI)—which was assessed using the growth index, where 1 point was given if volume of callus tissue was less than volume of the explant, 2 points were given if volume of callus tissue was approximately equal to volume of the explant, and 3 points were given if volume of callus tissue was greater than volume of the explant; this parameter was assessed in the final phase of callusogenesis (on the 20th day) as a percentage in relation to total number of explants [43]. The total water content in calli was determined by changes in their weight. Callus growth rate (CGR) was calculated as the difference between the fresh weight or volume of the callus before transfer to a culture medium containing PEG (or medium without PEG for control samples) and the fresh weight or volume of the same callus after 10 days of cultivation, expressed as a percentage of the initial weight. The viability of callus cultures (VC) was assessed using the time of emergence of first necrotization signs (days) and the time of death of the culture within the 4 months of incubation.

### 4.3. Callus Cultures’ Biochemical Characteristics

To check how strongly the selected PEG doses affect the callus cells, their viability was evaluated. The viability of callus culture cells (VCC) was determined using the reduction of 2,3,5-triphenyltetrazolium chloride (TTC, Dia-M, Moscow, Russia), which is reduced to red water-insoluble formazan by the activity of dehydrogenases in a living cell [44]. The resulting red formazan was extracted from cells by incubation with 95% ethanol at 65 °C for 15 min. The formazan ethanol solution absorbance was measured at 485 nm using Bio-Rad SmartSpec Plus spectrophotometer. Extinction was calculated per 1 g of callus fresh weight.

Oxidative stress at the cell level accompanies all stressful effects. To identify whether intracellular oxidation occurs as a result of the action of water deficiency, the content of reactive oxygen species as well as the activity of lipid peroxidation and of antioxidant enzymes catalase and peroxidase were evaluated. To determine the content of superoxide anion (SA), 0.3 g of callus tissue was slightly loosened and incubated with NBT (Gerbu, Heidelberg, Germany) solution (1 mg cm^−3^) in 10 mM KH_2_PO_4_ (pH 7.8) in the dark [45] in a water bath at 37 °C for 1 h, washed with distilled water, and dried on filter paper. The resulting dark-blue formazan was extracted through grinding in a 2M KOH solution in DMSO (CDH, India) (1:1.167) according to Myouga et al. [46]. After centrifugation for 10 min at 12,000× *g*, the absorbance of supernatant was measured at 700 nm using a SmartSpec Plus spectrophotometer (Bio-Rad, Hercules, CA, USA). Extinction was calculated per 1 g of fresh weight. To determine the hydrogen peroxide (HP) content, 0.3 g of loosened callus was incubated with 3,3′-diaminobenzidine (DAB, Sigma, Burbank, CA, USA) (2 mg cm^−3^) in a 10 mM of Tris-acetate (pH 5.0) in the dark in a water bath at 37 °C for 5 h, according to [47]. Subsequently, the callus was washed with distilled water and dried on filter paper. The resulting brown pigment was extracted with 0.2 M HClO_4_. After centrifugation for 10 min at 12,000× *g*, the absorbance of the supernatant was measured at 450 nm using a SmartSpec Plus Bio-Rad spectrophotometer. HP content was determined using a calibration curve method. HP concentration was assessed in relation to the fresh callus weight. To measure the activity of lipid peroxidation (LP), the activity of catalase (CAT) and peroxidase (POD) enzymes, and to detect DHN, callus samples were frozen in liquid nitrogen and stored in a cryostat at −70 °C. LP activity was determined by assessing the content of products (MDA-eq) resulting from the reaction with thiobarbituric acid (TBA), as described in [48]. For this, 0.3 mg of callus was ground with 1.5 mL of 0.1% trichloroacetic acid (TCA, Panreac, Castellar del Vallès, Spain), and the mixture was centrifuged (12,000× *g* for 15 min). Then 2 mL of 0.5% TBA (Dia-M, Russia) in 20% TCA was added per 1 mL of the resulting supernatant, and the mixture was incubated in a boiling water bath for 30 min. The reaction was stopped by cooling, and the test tubes were placed on ice. The samples were centrifuged for 5 min at 12,000× *g,* and the absorbance of supernatant was measured at 532 and 600 nm. The MDA-eq content was calculated using the TBA extinction coefficient of 155 mM^−1^ cm^−1^ after subtracting the nonspecific absorbance measured at 600 nm and expressed as nM g^−1^ of fresh weight. To measure the enzyme activities, a frozen callus sample (0.5 g) was ground in liquid nitrogen with the addition of quartz sand and 2 mL of 0.2 M sodium-phosphate buffer (pH 7.0). After centrifugation, during the period of taking enzyme activity measurements, the supernatant was placed on ice in glass tubes and stored at +4 °C. The CAT activity was measured in 0.2 M sodium-phosphate buffer (pH 7.0) containing 11.5 mM hydrogen peroxide spectrophotometrically at 240 nm by the rate of decrease in the hydrogen peroxide content in the reaction medium using the hydrogen peroxide extinction coefficient ɛ_240_ = 0.036 mM^−1^·cm^−1^. CAT activity was expressed as the rate of hydrogen peroxide loss per 1 mg of the sample fresh weight: nM·sec^−1^·mg^−1^ of fresh weight. The POD activity was measured in 0.2 M sodium-phosphate buffer (pH 7.0) containing 10 mM hydrogen peroxide and 8 mM guaiacol (CDH, Delhi, India) spectrophotometrically at 470 nm by the rate of formation of the reaction product tetraguaiacol using the extinction coefficient of tetraguaiacol ɛ_470_ = 0.0266 mM^−1^·cm^−1^. POD activity was expressed as the rate of tetraguaiacol formation per 1 mg fresh weight of the sample: mkM·min^−1^·mg^−1^ fresh weight (U·min^−1^·mg^−1^ fresh weight). Enzyme activity measurements were carried out at a constant temperature of +25 °C.

### 4.4. RNA Isolation and RT-qPCR

The effect of water deficiency on the activity of dehydrin genes was assessed using RT-qPCR. Total RNA was extracted according to Verwoerd et al. [49], with slight modifications, by heat phenol extraction method. Fifty to one hundred and fifty mg of callus tissue was homogenized in liquid nitrogen using mortar and pestle. After grinding, 300 μL of phenol (Dia-M, Russia), 270 μL of extraction buffer (10 mM Tris-HCl and 1 mM EDTA; pH 7.4), and 30 μL of 10% SDS solution were added to each sample. Homogenates were collected in 2 mL tubes (Eppendorf, Framingham, MA, USA), incubated at 65 °C for 10 min, and then centrifuged at room temperature at 14,000× *g* for 10 min. A total of 300 μL of supernatant was collected, mixed, and vortexed with 150 μL of chloroform-isoamyl alcohol solution (24:1) with 150 μL of phenol. After centrifugation at room temperature for 10 min at 14,000× *g*, 250 μL of supernatant was collected, mixed with 200 μL of chloroform-isoamyl alcohol solution (24:1), and vortexed (this procedure was repeated twice). The sample was centrifuged at room temperature at 14,000× *g* for 10 min, then 200 μL of supernatant was collected and mixed with 2.5 volumes of 96% ethanol and NaCl (to the concentration of 0.2 M), vortexed gently, and stored at –20 °C for 18 h. The samples were centrifuged at 14,000× *g* for 10 min at 4 °C, and RNA pellets were washed twice with 70% ethanol, dried, and resuspended in nuclease-free water.

For cDNA synthesis, a sample containing 400 ng of total RNA pretreated with DNaseI (Thermo Scientific, Waltham, MA, USA) was used. The synthesis was performed using random hexamer primers (Thermo Scientific) and RevertAid H Minus M-MuLV reverse transcriptase (Thermo Scientific, USA) according to the manufacturer’s protocol. The RT-qPCR was performed with qPCRmix-HS SYBR (Evrogen, Moscow, Russia) using C1000 Thermal Cycler CFX96 Real-Time PCR Detection System (Bio-Rad, USA) according to the following protocol: heating to 50 °C for 2 min, first denaturation cycle (95 °C, 3 min), and 36 amplification cycles (95 °C, 20 s; 60 °C, 30 s; and 72 °C, 30 s). The *ACTIN-1* gene was used as a reference [50]. The sequences of primes used in the current study are listed in Table 5.

RT-qPCR expression data were estimated and normalized using 2^−ΔΔCt^ method. For heatmap visualization, the expression values were log_2_-transformed and the heatmap is visualized using ComplexHeatmap R package [51].

### 4.5. DHN Detection

Callus samples of 0.5 g were used for total protein extraction. The samples were ground in liquid nitrogen with quartz sand in 2.5 mL buffer containing 100 mM Tris-HCl (pH 7.4–7.6), 0.1% sodium dodecyl sulfate (SDS), 12 mM β-mercaptoethanol, 1 mM phenylmethylsulfonyl fluoride (PMSF), and insoluble polyvinylpyrrolidone (PVPP, 10% of sample weight). After centrifugation (18,000× *g*, 10 min), the protein was precipitated from the resulting supernatant by addition of five volumes of ice-cold acetone (−20 °C, 8500× *g*, 10 min). The total cellular protein pellet was dissolved in sample buffer containing 0.125 M Tris-HCl (pH 6.8), 10% SDS, 10 mM β-mercaptoethanol, 1 mM ethylenediaminetetraacetic acid (EDTA), and 20% glycerol, followed by heating at 97 °C for 3 min in a water bath and centrifugation at 12,000× *g* for 10 min. The protein concentration in the resulting samples was determined using a QubitTM fluorimeter (Invitrogen, Carlsbad, CA, USA) according to the instructions using a set of reagents (Quant-iT Protein Assay kits, Invitrogen, USA). To detect DHNs and to determine the DHN content, 15 μg of protein from each sample was separated electrophoretically in a polyacrylamide gel with SDS and transferred to a nitrocellulose membrane in a mini-Protean III system (BioRad, USA) in accordance with the manufacturer’s instructions. The amount of protein applied was further normalized using densitometry after staining the gel with Coomassie Brilliant Blue G-250. Proteins were detected using primary antibodies. Polyclonal antibodies against DHNs were used (AS07 206, Agrisera, Vännäs, Sweden; dilution 1:500). Antibodies were visualized using alkaline phosphatase-conjugated secondary antibodies (Sigma, USA) using BCIP/NBT substrate (Gerbu, Heidelberg, Germany). The molecular weights (Mr) of the identified DHNs were determined using protein markers PageRuler Unstained Protein Ladder (Thermo Scientific, USA) using Image Lab software (version 5.2) (Bio Rad, USA). The DHN content was assessed by the intensity of protein spot staining with the subtraction of background staining on the digital image of the membrane using Image Lab software (version 5.2) (Bio Rad, USA). The content of every single DHN in a sample was expressed in arbitrary units in relation to the total dye intensity of this protein in all samples on the studied membrane, which was taken as one [25].

### 4.6. Statistical Analysis

Measurements were carried out with at least three replicates, and the arithmetic mean and standard error of the mean were calculated. The normality of the sample distribution was assessed according to the Kolmogorov–Smirnov criterion, and Levene’s test was used for equal variation assessment. The reliability of the differences in the sample with a normal distribution was assessed using the t-test; in other cases, the nonparametric Mann–Whitney test was used. Correlation and regression analyses of gene expression data were conducted using ggpubr R package [52]. For the regression analysis, we used the data obtained via ddCt method (LogFC) for transcripts and log fold changes of DHN protein content in samples after treatment. The intensity of the correlation connection between indexes of growth, oxidation, and protein accumulation was assessed using Spearman’s non-parametric criterion in Exel and Statistica software, version 10.0 (*p* < 0.05). The reference [52] is cited in the Supplementary Materials. We used the SigmaPlot and Statistica programs.

## 5. Conclusions

In this study, we assessed the effect of a low level of water deficiency on the accumulation of dehydrins in the callus cells obtained from the buds and branches of Scots pine trees. It was possible to show for the first time that a low level of water deficiency causes the accumulation of not only high-molecular-weight dehydrin Mr 72 kD but also dehydrins Mr 38 and 27 kD. According to the results of our study, a decrease in the growth rate of cultures and an increase in the activity of lipid peroxidation were associated with a decrease in the water content in cells. In most cultures, the activity of the antioxidant enzyme peroxidase also increased. This indicates that the low level of water deficiency used had an effect on the callus cultures that was associated with intracellular oxidation. Under the action of a low level of water deficiency, the accumulation of Mr 72 and 27 kD dehydrins in bud-derived callus cells was statistically correlated with the development of lipid peroxidation. Thus, the accumulation of dehydrins Mr 72 and 27 kD in the callus cultures of buds may be a reaction to intracellular lipid oxidation, which develops as a result of the influence of a low level of water deficiency. It can be assumed that dehydrins Mr 72 and 27 kD, when acting in callus cells under a low level of water deficiency, play the role of antioxidants. The accumulation of dehydrins in the cells of callus cultures, which initially differ significantly in their viability, speaks in favor of the universality of the accumulation reaction of these proteins. The study also shows that callus cultures of coniferous trees are suitable for studying resistance factors to abiotic stressors. However, further study of the differences in the accumulation of dehydrins in trees differing in genotypes, as well as the use of higher concentrations of PEG or other methods of modeling water deficiency more closely related to the natural environment, can be used to expand our knowledge about the identified dehydrins of the species Scots pine under the action of water deficiency.

## Figures and Tables

**Table 1:** The callus growth rate (CGR) (the change in callus volume during cultivation; DAP—days after planting).

Number of the Tree	Source of Explant	15 DAP, mm^3^	30 DAP, mm^3^	45 DAP, mm^3^
1	buds	60	119	200
	branches	33	85	179
2	buds	55	135	337
	branches	35	102	148
3	buds	132	285	581
	branches	31	209	832
4	buds	93	143	328
	branches	23	245	324
5	buds	25	100	271
	branches	43	91	290

**Table 2:** The callus growth rate (CGR) (the rate of callus growth measured after 15 days of cultivation; percentage of mass gain; DAP—days after planting).

Number of the Tree	Source of Explant	15–30 DAP	30–45 DAP	45–60 DAP
1	buds	197	167	208
	branches	261	210	250
2	buds	244	250	172
	branches	293	145	186
3	buds	216	204	206
	branches	674	398	269
4	buds	154	230	199
	branches	1078	132	255
5	buds	400	270	153
	branches	214	319	185

**Table 3:** The viability of callus cultures (VC) (observations over 4 months of cultivation).

Number of the Tree	Source of Explant	First Detection of Necrotization Areas, Days	Death of the Culture, Days
1	buds	14–35	38–84
	branches	14–35	44–84
2	buds	14–35	44
	branches	23–35	44–48
3	buds	22–29	-
	branches	26–120	-
4	buds	22–38	-
	branches	50–120	-
5	buds	35–38	-
	branches	35–120	-

**Table 4:** The callus growth rate (CGR) on culture medium with added PEG (mass gain percent).

Number of the Tree	Source of Explant	Control	5% PEG	8% PEG
1	buds	153	137	139
	branches	179	167	142
2	buds	145	127	120
	branches	133	133	132
3	buds	173	146	139
	branches	161	150	137
4	buds	156	145	151
	branches	166	151	145
5	buds	173	152	144
	branches	154	149	135

**Table 5:** Sequences of primers used in the study.

ID	Gene	Forward Primer	Reverse Primer
GQ339779.1	*ACT-1*	ACGGAGGCACCACTTAACCC	ATCCGTCAGATCACGCCCAG
JQ969658.1	*DHN1*	TGCCTGAGAGCATTGATGGGA	TTGACCGAACACTCAGGACCC
EU394116.1	*DHN2*	CAATGCCCAGGTTACGGC	AGCTGTTTGTGCGGTGAAGC
FJ201358.1	*DHN3*	AAAGCAGTGTTTGCGGTCAGC	TCCATGCTCCTCACCCAAGC
FJ201392.1	*DHN4*	GGGAAGAAGCCGGGAATGGTA	CTGACCGCCACACTGCTTTC
AJ512365.1	*DHN6*	GCTGTCACCCTGGTCGTGTA	TCCCGGCAGCTTCTGTTTGA
AJ512366.1	*DHN7*	ATGGCGGAAGAGCAACAGGA	GCTGACCGCAACACTGCTTT
AJ512367.1	*DHN8*	ATGGCGGAAGAGCAACAGGA	AGTCCGAGGAGGACCCTGAT
FJ201521.1	*DHN9*	AAGCACCTGAGCACCAGGAC	GCTTGCTTTCCCTCCTCTTCCT

## Data Availability

The original contributions presented in the study are included in the article/Supplementary Material; further inquiries can be directed to the corresponding author.

## Supplementary Materials

The following supporting information can be downloaded at: https://www.mdpi.com/article/10.3390/plants13192752/s1, Table S1: The start of callusogenesis; Table S2: The frequency of callus induction (FCI) assessed at different cultivation days (DAP—Days After Planting); Table S3: The Callusogenesis Intensity (CI) (growth index measured at the day 22 of cultivation), percent; Table S4: Meanings of R and p for equations of the dependencies of investigated indexes of callus cultures at control conditions; Table S5: Meanings of R and p for equations of the dependencies of investigated indexes of callus cultures at 5% PEG; Table S6: Meanings of R and p for equations of the dependencies of investigated indexes of callus cultures at 8% PEG; Table S7: Meanings of R and p for equations of the dependencies of indexes of callus cultures, which were changed under the influence of 5% or 8% PEG; Figure S1: Source of explants used for callus initiation, the explants planted on a growth medium, and the emergence of calli on explants; Figure S2: The appearance of callus cultures after cultivation with PEG (5 or 8%) added to growth medium and control (untreated with PEG) callus samples. Five replicates are shown; Figure S3: The correlation of VCC with culture growth rates of t1–t5 under the control conditions. Correlation and regression analyses of data were conducted using ggpubr R package [52]; Figure S4: Linear regression analysis for transcripts and changes of DHN 72 protein content in samples after PEG treatment [52]; Figure S5: Linear regression analysis for transcripts and changes of DHN 38 protein content in samples after PEG treatment [52]; Figure S6: Linear regression analysis for transcripts and changes of DHN 27 protein content in samples after PEG treatment [52].
