# Supplementary material for: The Influence of Water Deficit on Dehydrin Content in Callus Culture Cells of Scots Pine
Source: Plants (Basel). 2024 Sep 30;13(19):2752. doi: 10.3390/plants13192752 (PMC11479203; doi:10.3390/plants13192752)
Supplement: Supplementary file 1 [file plants-13-02752-s001.zip › Table S1 Suppl.pdf]

Table S1. The start of callusogenesis

	Source of explant	The day callus formation first appeared[1]
1	buds	2
	branches	5
2	buds	2
	branches	5
3	buds	2
	branches	5
4	buds	2
	branches	5
5	buds	2
	branches	5

[1] detected from the day of cultivation start
