# Supplementary material for: The Influence of Water Deficit on Dehydrin Content in Callus Culture Cells of Scots Pine
Source: Plants (Basel). 2024 Sep 30;13(19):2752. doi: 10.3390/plants13192752 (PMC11479203; doi:10.3390/plants13192752)
Supplement: Supplementary file 1 [file plants-13-02752-s001.zip › Table S2 Suppl.pdf]

Table S2. The frequency of callus induction (FCI) assessed at different cultivation days (DAP – Days After Planting).

Number of the tree	Source of explant	The percentage of callusogenesis assessed at:					
		2 DAP	3 DAP	5 DAP	10 DAP	15 DAP	20 DAP
1	buds	15,8	30,8	72,9	77,4	80,4	98,5
	branches	0	0	1,9	30,6	46,8	60,6
2	buds	10,1	17,6	66,9	81,8	81,8	98,6
	branches	0	0	8,2	47,9	55,6	85,4
3	buds	10,4	20,8	68,8	88,0	88,0	99,2
	branches	0	0	4,4	36,6	57,8	96,3
4	buds	8,8	13,5	66,9	85,3	95,3	100,0
	branches	0	0	1,8	16,1	25,6	75,0
5	buds	7,1	12,8	37,6	52,2	53,9	99,3
	branches	0	0	5,6	19,9	28,6	75,8

The frequency of callus induction (FCI) on the explants varied both between individual trees and between explants of different origin from each tree (Table 2). FCI at the end of callusogenesis phase (day 20) was 98.5% for bud explants of t1, 98.6% for t2, 99.2% for t3, 100% for t4, and 99.3% for t5. While callus on branches explants of t1 by the day 20 was formed only in 60.6% of cases, t2 - in 85.4%, t3 - 96.3%, t4 - 75.0%, t5 - 75.8% of cases. FCI for explants of different origins and genotypes varied significantly depending on specific intermediate dates for assessing this parameter – at the days 2, 3, 5, 10 and 15 of cultivation (Table 2). Noteworthy is the low FCI on the t1 branches explants (60.6% of cases by the day 20 of cultivation).
