# Supplementary material for: The Influence of Water Deficit on Dehydrin Content in Callus Culture Cells of Scots Pine
Source: Plants (Basel). 2024 Sep 30;13(19):2752. doi: 10.3390/plants13192752 (PMC11479203; doi:10.3390/plants13192752)
Supplement: Supplementary file 1 [file plants-13-02752-s001.zip › Table S3 Suppl.pdf]

Table S3. The Callusogenesis Intensity (CI) (growth index measured at the day 22 of cultivation), percent.

Number of the tree	Source of explant	No callusogenesis	1 point	2 point	3 point
	buds	2,5	24,7	22,2	50,6
	branches	37,6	17,7	25,9	18,8
2	buds	2,1	30,2	21,9	45,8
	branches	28,5	14,3	28,6	28,6
3	buds	1,6	11,3	25,8	61,3
	branches	7,2	23,2	31,9	37,7
4	buds	2,2	17,4	23,9	56,5
	branches	39,7	19,2	31,5	9,6
5	buds	0,0	15,4	38,5	46,1
	branches	40,4	26,9	17,3	15,4

It was found that callusogenesis intensity (CI) for all studied trees was higher on plant material obtained from buds (Table 3). The primary callus significantly exceeded the size of the explant on which it was induced in over 45% of cases for bud explants of t2 and t5, and in over 50% of cases for bud explants of t1 and t4, and in over 60% of cases for tree t3. The branches explants of t1 were characterized by a relatively equal distribution of the number of explants with low and high CI values. A significantly higher number of explants were included in the group with an average CI value. For t2 the number of explants with medium and high CI values was found to be the same and was higher than the number of explants with low CI values. The branches explants of t3 were characterized by a relatively equal distribution of the number of explants with low, medium and high CI values. Wherein calli with a high growth index predominated (37.7%). On the contrary, the branches explants of t4 and t5 demonstrated the smallest number of calli with high IC values (9.6% and 15.4%, respectively). The largest number of calli induced from branchesof t4 and t1 had an average rating of CI - 2 points (31.5% and 25.9%, respectively), which indicates a lower callus growth rate. The material from branches of t5 was characterized by the lowest CI values. The study of the characteristics of growth and development of callus cultures allows us to conclude that there are also differences in these characteristics between samples obtained from the studied trees; the lowest CI values were detected for derivatives of t2 and t5 buds and derivatives of t4 and t5 branches.
