# Supplementary material for: The Influence of Water Deficit on Dehydrin Content in Callus Culture Cells of Scots Pine
Source: Plants (Basel). 2024 Sep 30;13(19):2752. doi: 10.3390/plants13192752 (PMC11479203; doi:10.3390/plants13192752)
Supplement: Supplementary file 1 [file plants-13-02752-s001.zip › Table S4 Suppl.pdf]

Table S4. Meanings of R and p for equations of the dependencies of investigated indexes of callus cultures at control conditions*

	CGR	WC	VCC	LP	CAT	POD	HP	SA	DHN 72	DHN 38	DHN 27
**control branches**											
CGR	-	0,070723	0,769728	0,555868	-0,00494	0,084357	-0,45216	-0,68143			
WC		-	0,19186847	-0,7041438	0,59421216	R=0,915481<br>p=0.037	R=-0,81490<br>p=0.037	-0,7266804			
VCC			-	0,48751596	0,54010078	0,13670652	-0,7163715	-0,4756398			
LP				-	-0,1735147	-0,5530874	0,24379273	0,15035385			
CAT					-	0,60058026	R=-0,75241<br>p=0.037	-0,2771041			
POD						-	-0,6893166	-0,7538669			
HP							-	0,73609223			
SA								-			
DHN 72									-		
DHN 38										-	
DHN 27											-
**control buds**											
CGR	-	R=0,857577<br>p=0.0048	0,101187	-0,38191	-0,2104	0,05628	-0,4842	-0,20301	0,127697	0,286769	0,363346
WC		-	0,38079407	-0,6568651	0,22052950	0,42816098	-0,7546450	-0,4739952	0,46510715	0,38148522	0,13418043
VCC			-	-0,1018598	0,48297985	0,92271044	-0,1840625	R=-0,92512<br>p=0.037	-0,1695239	-0,5640061	-0,6082471
LP				-	-0,7158306	-0,4268174	R=0,989915<br>p=0.016	-0,0068011	R=-0,72773<br>p=0.037	-0,6836950	-0,4919760
CAT					-	0,77539651	-0,6789629	-0,2540328	0,43181165	0,14895221	0,00629413
POD						-	-0,4747134	-0,7665406	0,07244313	-0,3101707	-0,3771795
HP							-	0,09949019	R=-0,70449<br>p=0.037	-0,6428172	-0,4403913
SA								-	0,05100593	0,47123243	0,75096707
DHN 72									-		
DHN 38										-	
DHN 27											-

* Underlined data mean that equations adequately described dependences of indexes and were significant at $p<0.05$. Red color means strong dependences
