# Supplementary material for: The Influence of Water Deficit on Dehydrin Content in Callus Culture Cells of Scots Pine
Source: Plants (Basel). 2024 Sep 30;13(19):2752. doi: 10.3390/plants13192752 (PMC11479203; doi:10.3390/plants13192752)
Supplement: Supplementary file 1 [file plants-13-02752-s001.zip › Table S5 Suppl.pdf]

Table S5. Meanings of R and p for equations of the dependencies of investigated indexes of callus cultures at 5% PEG*

	CGR	WC	VCC	LP	CAT	POD	HP	SA	DHN 72	DHN 38	DHN 27
**5% PEG branches**											
**CGR**	-	0,387018	0,828391	0,360749	0,117648	0,19205	-0,53872	-0,16879			
**WC**		-	-0,09711	R=-0,7057<br>p=0.037	0,599547	0,717677	-0,60182	R=-0,84697<br>p=0.037			
**VCC**			-	0,734183	-0,43299	-0,38204	-0,08846	0,078709			
**LP**				-	-0,5726358	-0,59613	0,142486	0,689613			
**CAT**					-	R=0,96711<br>p=0.016	-0,6567	-0,13797			
**POD**						-	-0,79419	-0,27835			
**HP**							-	0,188743			
**SA**								-			
**DHN 72**									-		
**DHN 38**										-	
**DHN 27**											-
**5% PEG buds**											
**CGR**	-	R=0,92718<br>p=0.037	0,222081	-0,41603	0,571647	0,357203	-0,51386	0,44973	0,322575	-0,37425	-0,14639
**WC**		-	0,332524	-0,43292	0,709433	0,461703	-0,60626	0,105978	0,165612	-0,62262	-0,38264
**VCC**			-	0,65387717	0,71943863	R=0,98773<br>p=0.037	-0,9308149	0,11884283	0,75266055	0,14834265	0,33442604
**LP**				-	-0,0141368	0,56882389	-0,3568833	0,20911680	0,65794057	0,64744405	0,79289573
**CAT**					-	0,74961278	-0,9025406	-0,0029159	0,44195411	-0,2213609	-0,2646632
**POD**						-	R=-0,95496<br>p=0.037	0,15670755	R=0,74667<br>p=0.037	0,05130521	0,29716197
**HP**							-	-0,1877361	-0,7183741	0,00935260	-0,1267023
**SA**								-	0,72671844	0,60713240	0,70516397
**DHN 72**									-		
**DHN 38**										-	
**DHN 27**											-

* Underlined data mean that equations adequately described dependences of indexes and were significant at $p<0.05$. Red color means strong dependences
