# Supplementary material for: The Influence of Water Deficit on Dehydrin Content in Callus Culture Cells of Scots Pine
Source: Plants (Basel). 2024 Sep 30;13(19):2752. doi: 10.3390/plants13192752 (PMC11479203; doi:10.3390/plants13192752)
Supplement: Supplementary file 1 [file plants-13-02752-s001.zip › Table S6 Suppl.pdf]

Table S6. Meanings of R and p for equations of the dependencies of investigated indexes of callus cultures at 8% PEG*

	CGR	WC	VCC	LP	CAT	POD	HP	SA	DHN 72	DHN 38	DHN 27
**8% PEG branches**											
CGR	-	0,677834	0,900241 p=0.037	0,005307	0,266961	-0,29453	0,051016	-0,28472			
WC		-	0,67828784	-0,3852371	0,36575223	0,34270479	-0,5134391	-0,6050484			
VCC			-	0,25521904	-0,0874195	-0,2743331	0,17269007	-0,5481865			
LP				-	-0,5083114	-0,2449796	0,49087315	0,15184202			
CAT					-	0,50620517	-0,7595932 p=0.037	0,47867899			
POD						-	-0,9266671 p=0.037	0,08345823			
HP							-	-0,1131206			
SA								-			
DHN 72									-		
DHN 38										-	
DHN 27											-
**8% PEG buds**											
CGR	-	0,786363	0,74963	-0,23207	0,211445	0,102767	-0,95415	0,372497	0,861639	0,465772	0,334359
WC		-	0,41338558	-0,7491352 p=0.037	0,37638659	-0,4090359	-0,7878741	-0,0823800	0,44548571	-0,0670281	-0,2883635
VCC			-	0,28046999	-0,0452842	0,33009262	-0,7239283	0,87145663 p=0.037	0,89719327	0,66683591	0,39990611
LP				-	-0,4002276	0,73048570	0,25714324	0,69734094	0,21762118	0,58854853	0,67900291
CAT					-	0,28213233	-0,4751232	-0,2572337	-0,2055156	-0,6266858	-0,4230490
POD						-	-0,2253825	0,54914405	0,25290956	0,31216265	0,58627281
HP							-	-0,3477599	-0,7398858	-0,2647690	-0,1855916
SA								-	0,71958004 p=0.037	0,74006395 p=0.037	0,54429570
DHN 72									-		
DHN 38										-	
DHN 27											-

* Underlined data mean that equations adequately described dependences of indexes and were significant at $p<0.05$. Red color means strong dependences
