# Supplementary material for: The Influence of Water Deficit on Dehydrin Content in Callus Culture Cells of Scots Pine
Source: Plants (Basel). 2024 Sep 30;13(19):2752. doi: 10.3390/plants13192752 (PMC11479203; doi:10.3390/plants13192752)
Supplement: Supplementary file 1 [file plants-13-02752-s001.zip › Table S7 Suppl.pdf]

Table S7. Meanings of R and p for equations of the dependencies of indexes of callus cultures, which were changed under the influence of 5% or 8% PEG*

branches contr. vs 8% PEG	Δ CGR	Δ WC	Δ POD	Δ LP	Δ DHN 72	Δ DHN 27
Δ CGR	-	**R=-0,813** p=0,037				
Δ WC		-				
Δ POD			-			
Δ LP				-		
Δ DHN 72					-	
Δ DHN 27						-

buds contr. vs 5% PEG	Δ CGR	Δ WC	Δ POD	Δ LP	Δ DHN 72	Δ DHN 27
Δ CGR	-		**R=-0,852** p=0,037			
Δ WC		-				
Δ POD			-			
Δ LP				-	**R=0,952401** p=0,037	**R=0,812173** p=0,037
Δ DHN 72					-	
Δ DHN 27						-

buds contr. vs 8% PEG	Δ CGR	Δ WC	Δ POD	Δ LP	Δ DHN 72	Δ DHN 27
Δ CGR	-					
Δ WC		-				
Δ POD			-			
Δ LP				-	**R=0,879218** p<0,05	
Δ DHN 72					-	
Δ DHN 27						-

*A change in the index means a difference between the index value at control and after the PEG action; it is indicated by a sign Δ before the corresponding indicator. Data mean that equations adequately described dependences of indexes and were significant at $p<0.05$. Only adequately described dependencies are presented.
