# Supplementary figures and images for: The Influence of Water Deficit on Dehydrin Content in Callus Culture Cells of Scots Pine
Source: Plants (Basel). 2024 Sep 30;13(19):2752. doi: 10.3390/plants13192752 (PMC11479203; doi:10.3390/plants13192752)

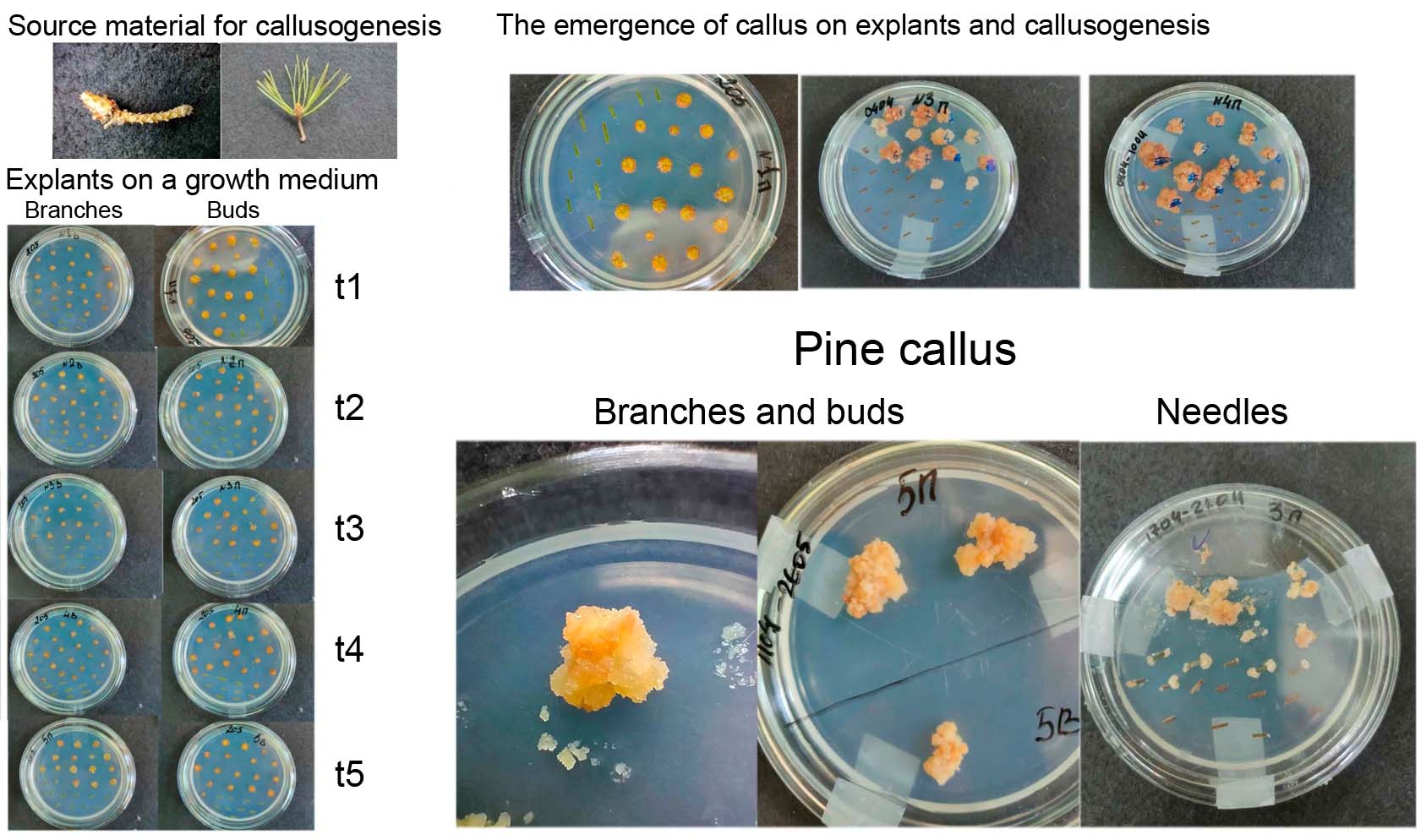

Supplement: Supplementary file 1 [file plants-13-02752-s001.zip › Figure S1.jpg]

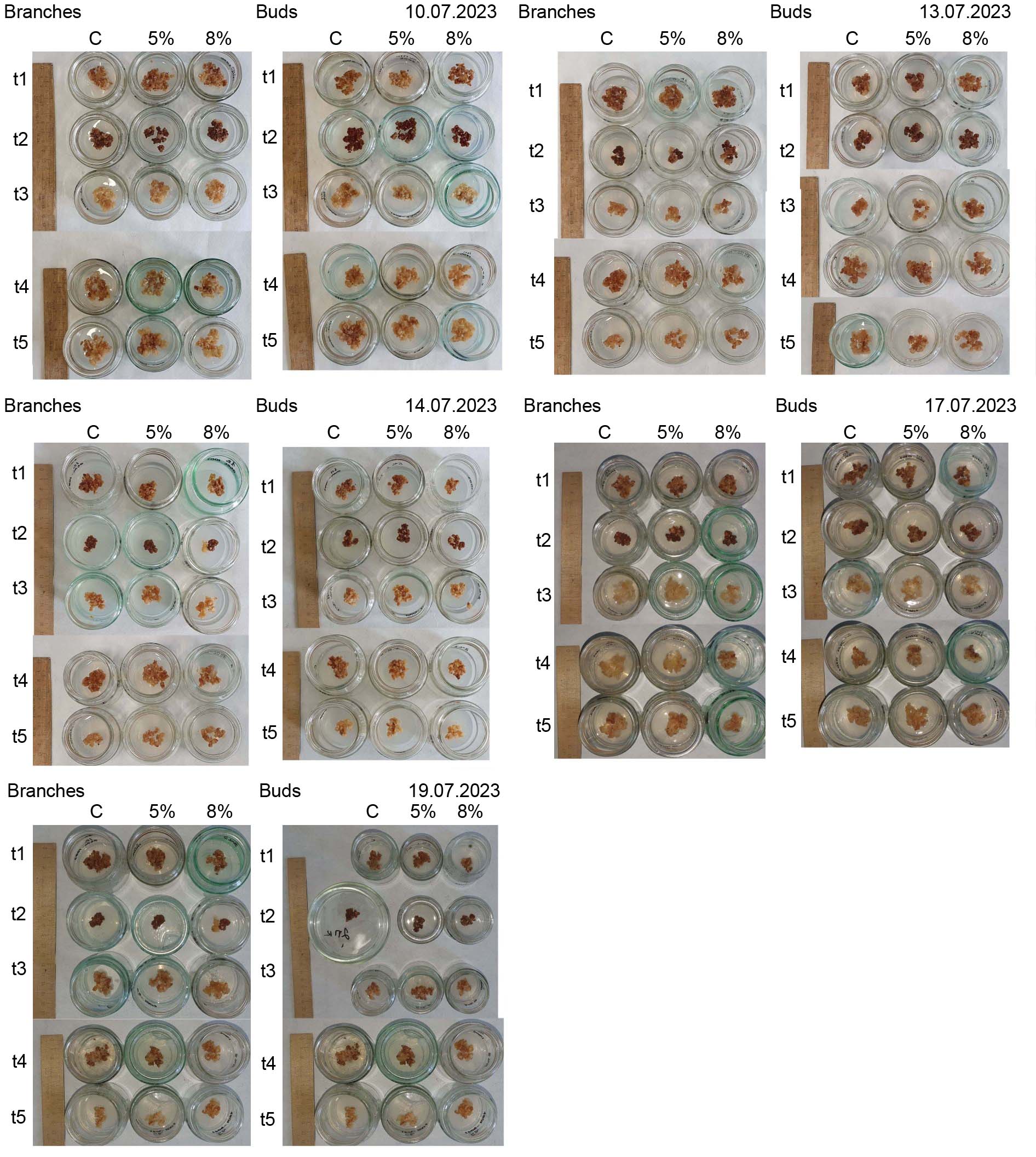

Supplement: Supplementary file 1 [file plants-13-02752-s001.zip › Figure S2.jpg]

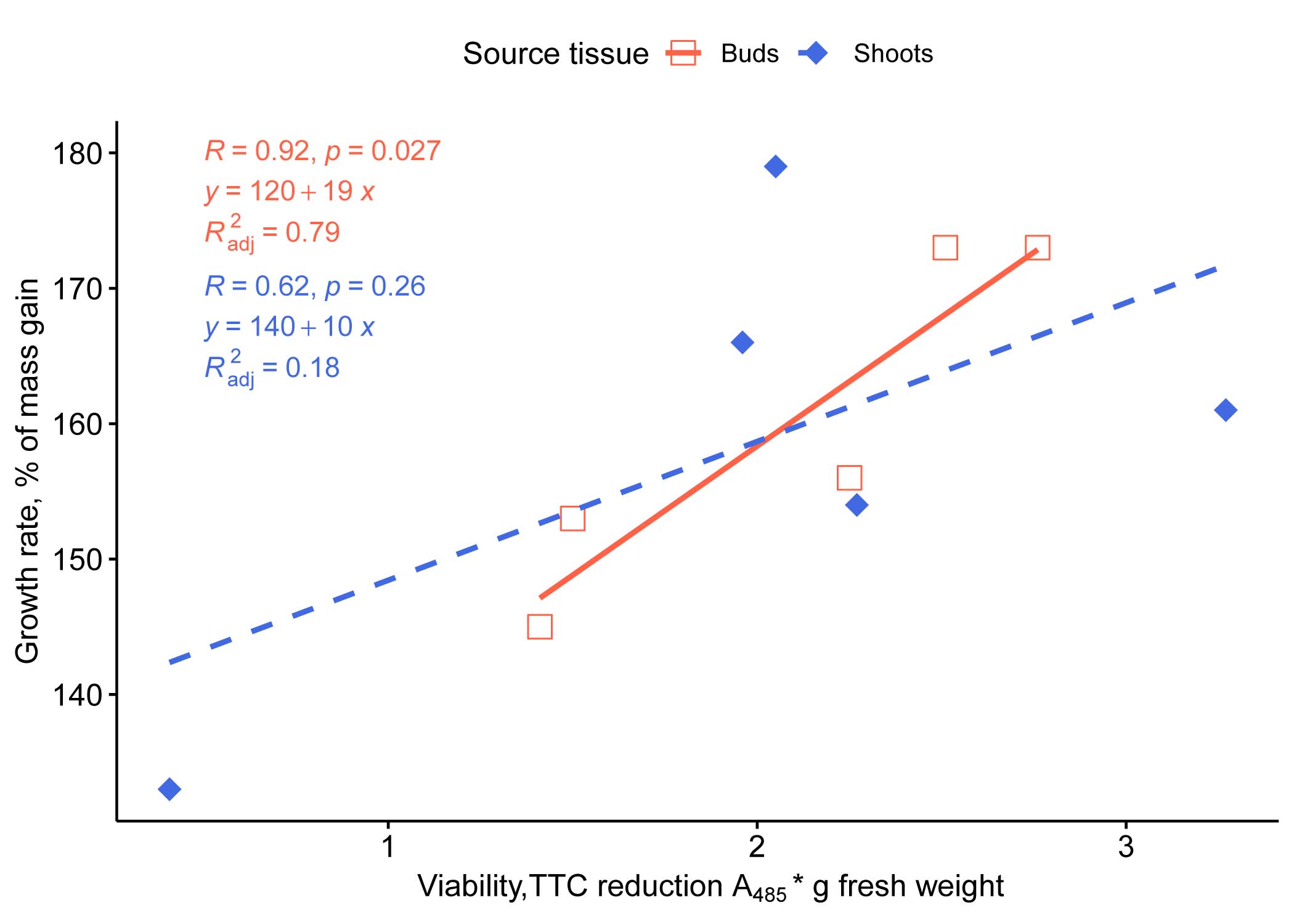

Supplement: Supplementary file 1 [file plants-13-02752-s001.zip › Figure S3.jpg]

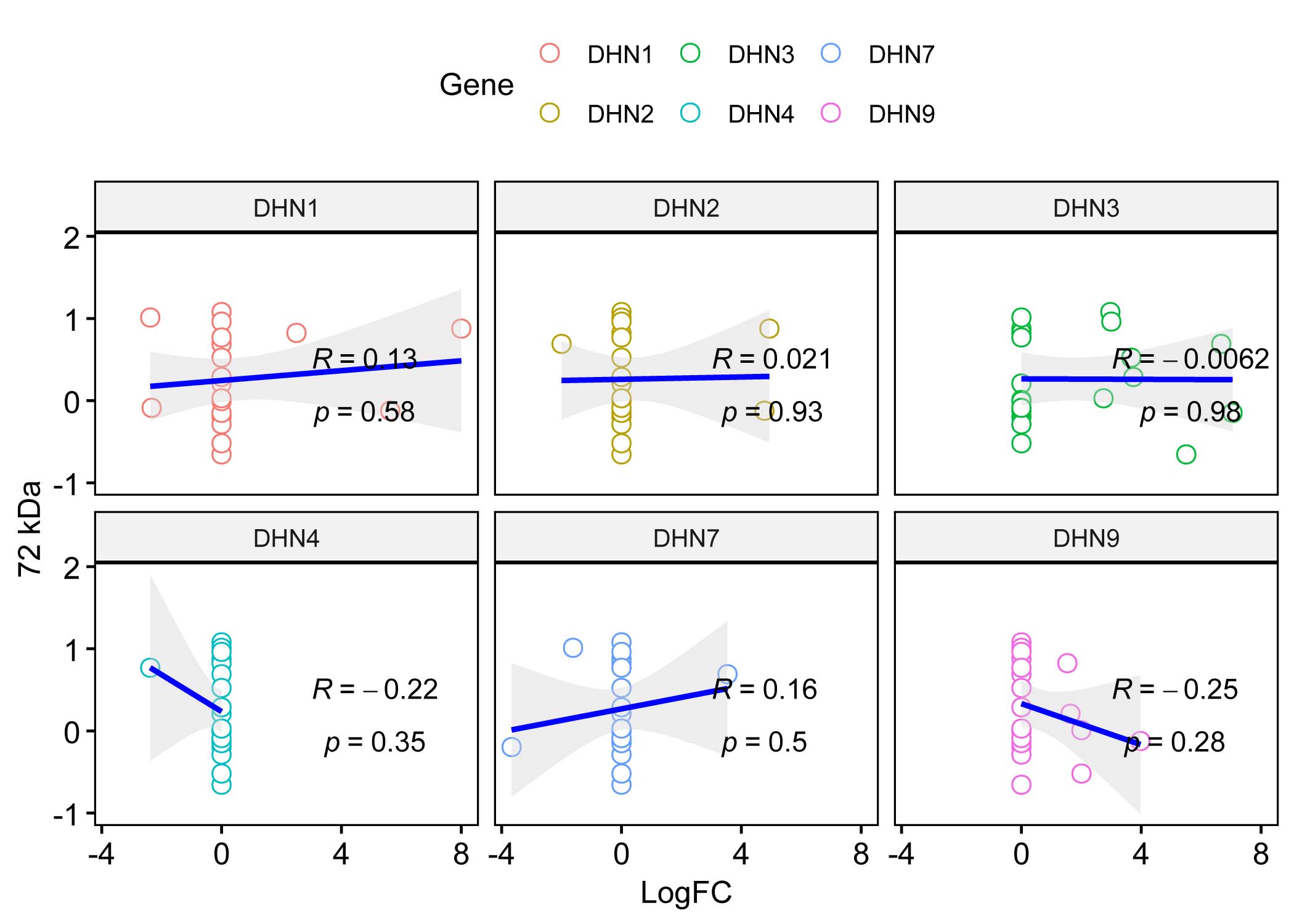

Supplement: Supplementary file 1 [file plants-13-02752-s001.zip › Figure S4.jpg]

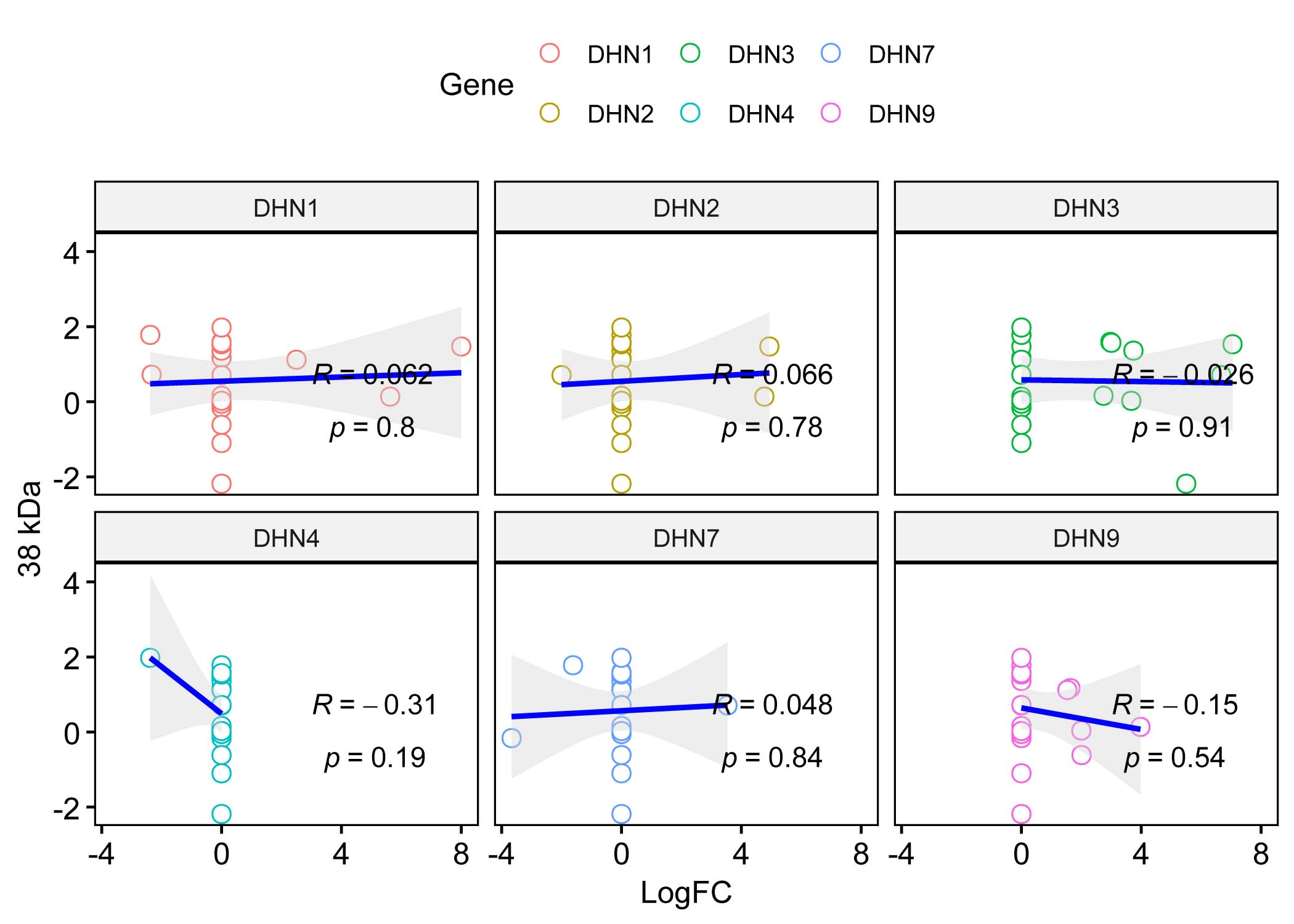

Supplement: Supplementary file 1 [file plants-13-02752-s001.zip › Figure S5.jpg]

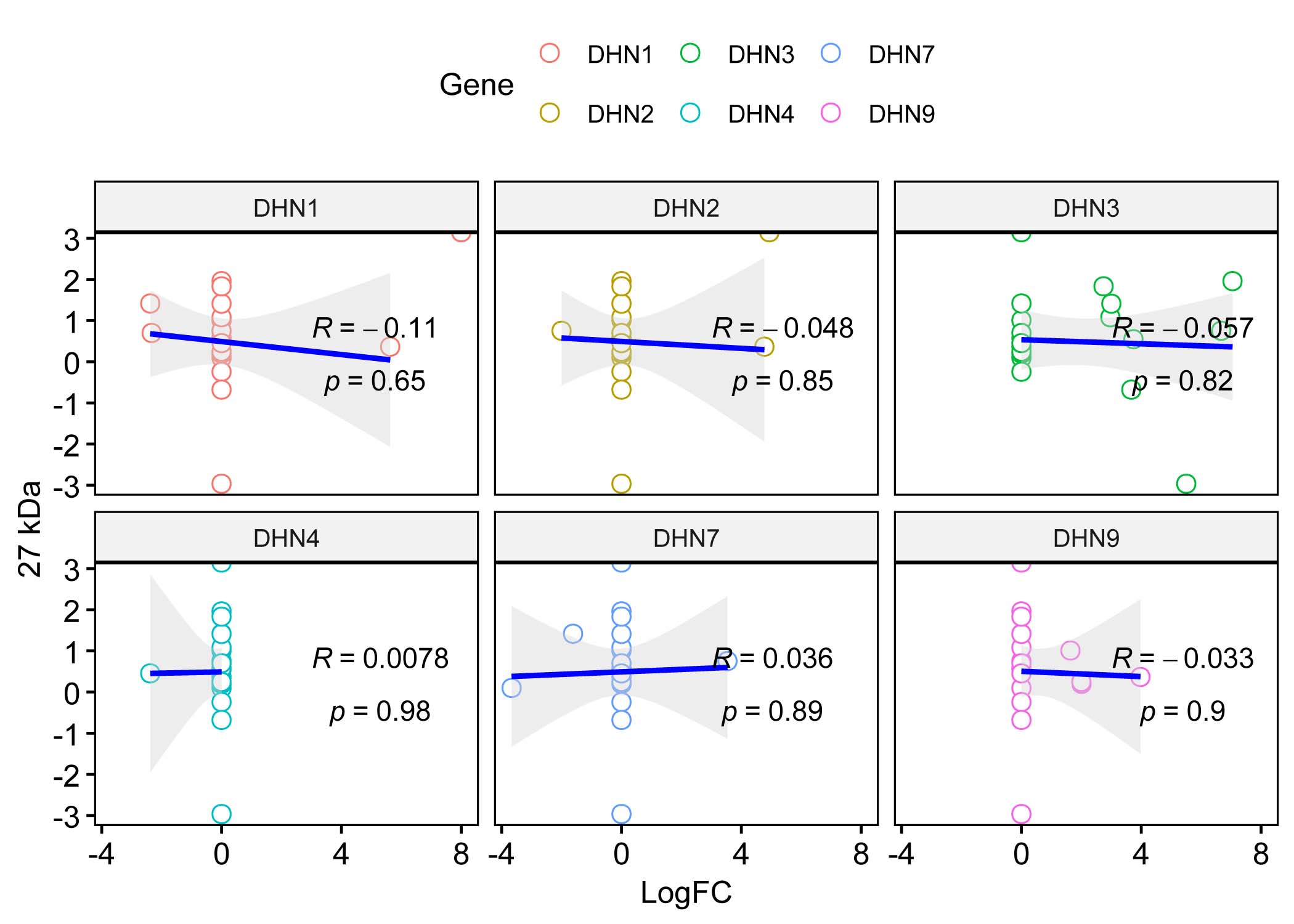

Supplement: Supplementary file 1 [file plants-13-02752-s001.zip › Figure S6.jpg]
